# Wine Quality Drivers: A Case Study on South African Chenin Blanc and Pinotage Wines

**DOI:** 10.3390/foods9060805

**Published:** 2020-06-18

**Authors:** Jeanne Brand, Valeria Panzeri, Astrid Buica

**Affiliations:** South African Grape and Wine Research Institute, Department of Viticulture and Oenology, Stellenbosch University, Stellenbosch 7600, South Africa; Valeria.Panzeri@icloud.com

**Keywords:** quality drivers, wine quality, Pinotage, Chenin Blanc, sensory evaluation, chemical fingerprint, high resolution mass spectrometry

## Abstract

The aim of the study was to propose a methodology for the elucidation of sensory and chemical wine quality drivers. The winners of the 2018 Top 10 Chenin Blanc and Top 10 Pinotage challenges and additional lower scoring wines for each cultivar were evaluated. The two sets underwent sensory profiling by Check-All-That-Apply (CATA) and a 20-point quality rating by industry experts in non-competition conditions and chemical fingerprinting by Liquid Chromatography-High Resolution Mass Spectrometry (LC-HRMS). Data were submitted to Correspondence Analysis (CA) and Principal Component Analysis (PCA) for sensory and chemistry, respectively, from which the standardised deviates were correlated to quality scores to identify the quality drivers. The results illustrated the possibility to determine positive and negative sensory quality drivers (attributes), while the identification of drivers for chemistry (ions) was challenging due to the number of signals generated by the fingerprinting technique. The configurations of the sensory and chemical spaces were compared, but the similarities were relatively low as measured by Regression Vector (RV) coefficients, 0.437 and 0.505 for Pinotage and Chenin Blanc, respectively. The proposed methodology can also be used to explore the sensory space of wine sample sets with the added dimension of the quality drivers which, in turn, highlight the experts’ opinions on what makes a winning wine.

## 1. Introduction

Wine is an aesthetic product [1] and its appreciation is considered mostly subjective, sometimes likened to art appreciation, especially when the terms used for its description are borrowed from art: “complexity”, “balance/harmony”, “development” [2].

Quality is described as a multidimensional concept [3] or multifaceted construct [4], difficult to define and therefore often avoided in scientific works [1]. As there is a lack of clear definition and/or defined parameters [1], quality is evaluated by proxy [1,3]. For wine, the three main proxies are color, taste and mouthfeel, and aroma (or flavor) [2,5]. As there is still uncertainty regarding its nature, quality is assessed through the proxies’ perception; they have different weight for the final quality score [6,7,8,9].

Quality manifests a range of intrinsic and extrinsic dimensions; between the two, intrinsic dimensions are perceived as more important [10]. Interestingly, “pleasure” is considered as one of the intrinsic dimensions, but subordinate to cognitive gustatory dimensions [1].

Quality evaluation can be carried out by experts, trained panels, and consumers alike, but it was demonstrated that these groups differ in their perception of quality and its intrinsic and extrinsic dimensions [11,12,13,14,15]. The context for evaluating quality is relevant. In competitions, it is usually carried out by tasters with experience; competitions have systems in place to check for consistency of judges and an audit system [16]. Evaluations can be carried out also out of competition, in which case the intent is different—profile, preference, quality assessment, etc. [2]. The results for quality evaluation depend on the panel, on the manner of evaluation, and on the information received prior to evaluation [1,17].

Generally, experts are preferred for wine quality assessment. This might be linked to the perceived “expert objectivity” [1]. Experienced judges are considered to have a more objective (and systematic) approach to wine tasting and a better technique, lexicon, acuity, and consistency developed in time through experience and exposure [2,3,13,14,15,18,19] but their emotional response cannot be ignored [20,21]. It was demonstrated that for experts, preference is correlated to the quality score [11,15], but inconsistencies are not unusual [15]. Experts also tend to use a combination of descriptive and hedonic terms when describing wines [11,22]. Moreover, experts may agree on which sensory characteristics drive quality, but can lack concept alignment (i.e., interpretation of quality as a concept) and ratings can vary according to each individual’s concept of quality [3,11].

Writing about wine quality also brings to mind difficulties in communicating the findings: while scientific writing should be clear and offer critical commentary of the product, wine descriptions are often used to evoke an emotional response or an image. The use of precise terms—for example, the ones included in flavor wheels [23,24]—could constitute a more objective and systematic approach when dealing with wine descriptors [2,25].

Despite all the issues related to defining and consistently evaluating quality, wines are often valued for their quality as perceived by professionals in the industry and every year many prestigious local and international competitions take place. Wine competitions play an important role in the wine industry. They are used by producers as a marketing tool and, to an extent, as a benchmarking exercise. Consumers obtain quality assurance, which can simplify purchase decisions and risk perception as these competitions are seen as a “trusted seal of approval” [26].

Researchers and the industry are keen to explain why certain wines win by correlating success in a competition (and by extent quality) to more objective measures such as chemical composition as independent measurement of the quality proxies—color, aroma (flavor), taste. Linking quality to chemistry was achieved with various degrees of success, depending on the types of chemical analysis and the statistical technique employed (Multilinear Regression Analysis/MRA [22], Partial Least Squares/PLS [27], PLS2 [11], Sequential and Orthogonalized PLS/SO-PLS [28]). In general, the literature reports on use of targeted chemical analyses (for example volatile aroma compounds [22,27]) and the use of statistical methods directed to the prediction of quality based on the chemistry and/or sensory data acquired.

Another way to elucidate why certain wines win is to consider sensory evaluation out of competition and identify descriptors associated with a quality wine. Quality is not simply equivalent to a wine’s sensory profile, but the profile acts as an indicator of, or a means through which quality is assessed. In this case, the methods reported in the literature varied from traditional quality assessments (Quantitative Descriptive Analysis/QDA) [22], Descriptive Analysis (DA) [4,11], to DA combined with expert-quality sorting [11] and sorting [18].

In this context, the aim of the current study was to establish a methodology that would assist researchers and the industry in determining the sensory and chemistry drivers of quality. The case studies for illustrating this methodology were the winners of the Top 10 Chenin Blanc Challenge and Top 10 Pinotage competition in 2018 in South Africa. To answer questions such as “What are the characteristics of the winning wines?” or “What do winning wines taste and smell like?” the aroma and taste sensory profiles of the wines that won were established. Check-all-that-apply (CATA, a multiple choice-based rapid sensory method) was used, followed by quality rating on a 20-point scale [9,29], resulting in the profiling of the wines and also the re-evaluation of their quality scoring in a non-competition setting. Wine fingerprinting by HRMS (an untargeted metabolomics-type approach) completed the samples’ characterization using an information-rich chemical technique. The results of the investigation could be used to explore the sensory space of a wine sample set with the added dimension of the quality drivers which, in turn, highlight the experts’ opinions on what makes a winning wine.

## 2. Materials and Methods

### 2.1. Wine Samples

The Pinotage set selected for the study was composed of the winners of the 2018 ABSA Top 10 Pinotage competition [30] wines and an additional five low scoring wines. The Chenin Blanc set contained the winners of the 2018 Standard Bank Top 10 Chenin Blanc Challenge [31] and an additional three low scoring wines. The wines were supplied by the Pinotage Association and the Chenin Blanc Association.

For both competitions, the top 10 wines are released in alphabetical order and are all considered winners. The details of the wines and the codes used are given in Table 1.

### 2.2. Sensory Evaluation

The tasting panel consisted of 27 industry experts (44% female, 65% male, average age 41) for the Chenin Blanc evaluation and 20 experts (37% female, 63% male, average age 43) for Pinotage, including winemakers and cellar masters that did not judge the wines during the competition. All the experts had more than 5 years’ experience working in the wine industry. More than 75% indicated 10 years of experience or more.

A single session was used to capture both sensory profiling data (using CATA) and quality scores. The panel was instructed to evaluate the nose/odor of the wines using a CATA list which was based on previous research and the Chenin Blanc [32] and Pinotage [33] aroma wheels respectively. The overall perceived quality of each wine sample was scored out of 20 according to the internationally used wine quality rating system [9,29]. All sensory data were captured on 10.1” Samsung galaxy tab A (2018) tablets, using the Compusense cloud software (Compusense Inc, Guelph, ON, Canada).

The sensory evaluation was performed in a well-ventilated and temperature-controlled room free of extraneous odors or noises. Wines were presented at 20 °C ± 2 °C in international tasting glasses (ISO NORM 3591, 1977). Each glass was coded with a random three-digit code and covered with a Petri as lid. The panel received 25 mL of each wine. Monadic sample presentation was applied. The order of sample presentation was randomized across judges according to a Williams Latin square design. Judges were not allowed to communicate with each other during the session. Information about the wines were only shared at the end of the sensory evaluation session.

### 2.3. Chemical Analysis

All solvents were MS purity and were purchased from Merck Chemicals Pty. Ltd. (Germiston, South Africa). HRMS coupled to liquid chromatography (LC-HRMS) was used for wine fingerprinting. The samples were analyzed by Ultra Performance Liquid Chromatography (UPLC, Waters Corporation) equipped with a Synapt G2 quadrupole time-of-flight mass spectrometer (Waters Corporation). The separation was carried out on an Acquity UPLC HSS T3 column (1.8 μm internal diameter, 2.1 mm × 100 mm, Waters Corporation) using 0.1% formic acid (mobile phase A) and acetonitrile (mobile phase B) and a scouting gradient over 10 min. Flow rate was 0.3 mLmin^−1^ and the column temperature 55 °C. The injection volume was 2 μL and the samples were injected directly without pre-treatment. Mass calibration was performed according to the manufacturer’s procedure. The MS was operated in both positive and negative mode, and the total number of features acquired as RT_m/z was 1466 for each sample. The software is directly integrated with SIMCA-P (SIMCA 14.1, Umetrics, Sweden) and the statistical algorithms are directly applied to the processed datasets [34].

### 2.4. Statistical Data Analysis

Data obtained from quality scoring were subjected to one-way Analysis of Variance (ANOVA). When a significant ANOVA result was obtained (at *p* < 0.05) the Fisher’s LSD post-hoc test was applied to perform pairwise comparisons of the wines (XLSTAT 2018, Addinsoft SARL, New York, NY, USA).

Contingency tables containing the CATA data were constructed by counting the number of citations for each attribute across the judges for every wine sample (Microsoft Excel 2016, Microsoft Corporation, Redmond, Washington, USA). The attributes were tabulated as variables in the columns and the wine samples as objects in the rows. The intersection of a row and column represented the number of times that the attribute in the corresponding column was cited by all the judges to describe the wine in the corresponding row.

The contingency tables were submitted to heatmap analysis which included Hierarchical Cluster Analyses (HCA) Chi-square tests and Cochran’s Q tests. Attributes identified as significant were subjected to Correspondence Analysis/CA (XLSTAT 2018, Addinsoft SARL, New York, NY, USA). Pearson’s correlation coefficients between the standardised deviates (Statistica 13, TIBCO Software Inc., Palo Alto, CA, USA), obtained from the CATA analysis, and the quality scores were calculated to identify negative and positive quality drivers [18].

For the chemistry data, Principal Component Analysis (PCA) and Hierarchical Cluster Analysis (HCA) were applied in order to find natural configurations in the data according to treatments and samples by grouping/clustering. The variables with the highest squared cosines for the first three dimensions were considered for variable selection. Regression vector (RV) coefficients between the CA, performed on the sensory data, and the PCA, performed on the chemical data, was calculated using the first three dimension of the CA and PCA outputs (XLSTAT 2018, Addinsoft SARL, New York, NY, USA).

## 3. Results

### 3.1. Quality Rating in Non-Competition Conditions

**Figure 1:**
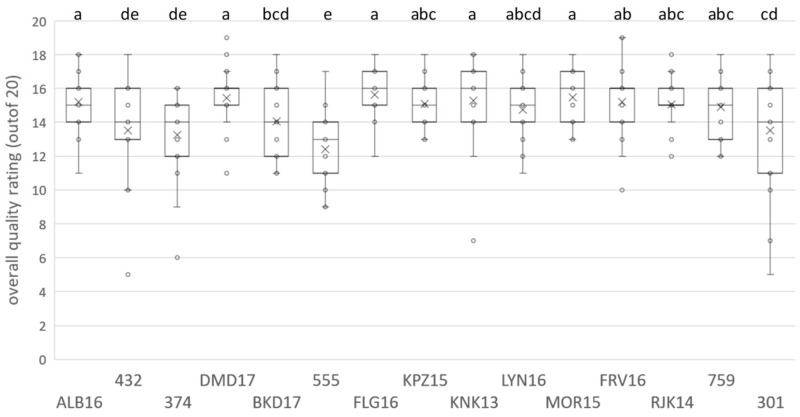
Box-and-whiskers plot illustrating the analysis of variance (ANOVA) results obtained for the overall quality rating for the Pinotage wines evaluated, including individual rating and mean marker. Letters indicate significant differences (*p* < 0.05). The codes are as specified in the text.

**Figure 2:**
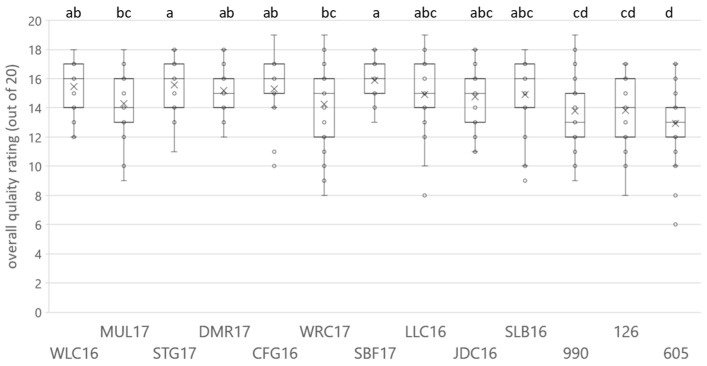
Box-and-whiskers plot illustrating the ANOVA results obtained for the overall quality rating for the Chenin Blanc wines evaluated, including individual rating and mean marker. Letters indicate significant differences (*p* < 0.05). The codes are as specified in the text.

The results for the Pinotage (Figure 1 and Table S1) and Chenin Blanc (Figure 2 and Table S2) wine sets indicated that the quality evaluation in non-competition conditions resulted in a relative broad distribution of scores. In one case, a competition low scoring wine (Pinotage wine 759) scored higher than two winners (LYN16 and BKD17), although the result was not statistically significant. For both sets, a continuum of scores was observed and only the lowest scoring wines were statistically placed in different groups compared to the highest. This difference was more evident in the case of Chenin Blanc (Table S2), possibly due to the lower number of samples in the set and not to the range of scores obtained, with average min-max 12.421–15.789 and 12.926–15.889 for Pinotage and Chenin Blanc, respectively.

### 3.2. Sensory Space and Sensory Drivers of Quality

Generally, the distribution of scores was narrower for higher scored wines. None of the wines obtained the maximum of 20 points; DMD17 and FRV16 from the Pinotage set scored the highest with 19 points. The lowest points were also in the Pinotage set, 5 for wine 432; one of the high scoring wines, KNK13, had one very low score of 7 points. In the Chenin Blanc set, 18 points was the highest score and seven of the wines attained it, while the lowest was 7 points for wine 605.

**Figure 3:**
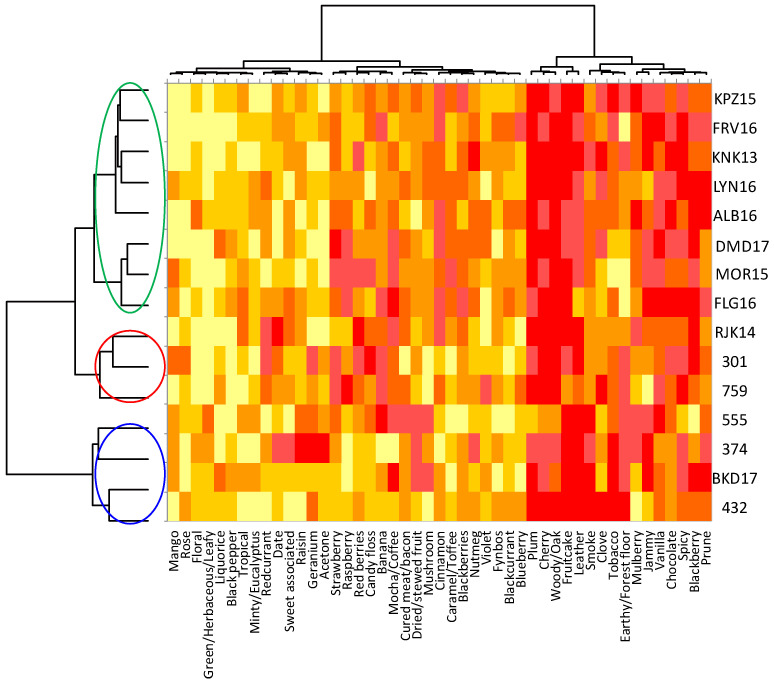
Heatmap associated with the results of the check-all-that-apply (CATA) task for the Pinotage wines evaluated. The dendrogram on the left corresponds to the Hierarchical Cluster Analysis (HCA) on the CATA results. Wine codes as indicated in the text.

**Figure 4:**
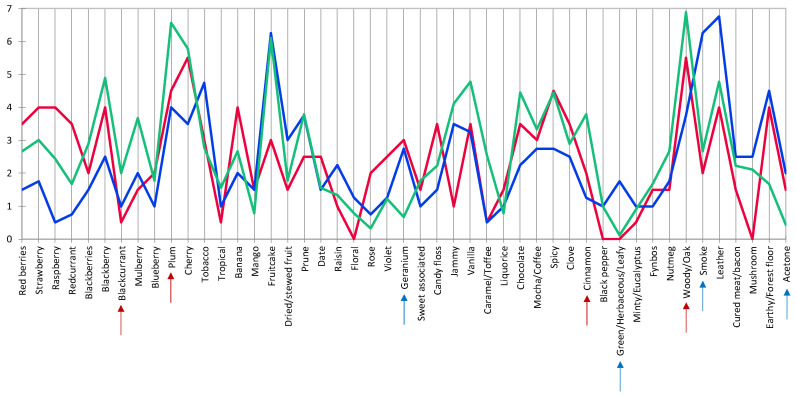
HCA cluster profile plot for Pinotage. The blue and red arrows indicate negative and positive drivers of quality, respectively. The color of the graph lines corresponds to the clusters indicated in Figure 3.

A general view of the sensory space for the Pinotage set could be observed on the heatmap (Figure 3). Notes of “acetone” are typical of an older style of this wine and it came as no surprise when this attribute was one of the negative drivers of quality, alongside “smoky”, “geranium” and “green/herbaceous/leafy” (Figure 4). “Blackcurrant”, “plum”, “cinnamon” and “woody/oak” were identified as positive drivers of quality (Figure 4). Visually, the frequency of citation of the attributes seemed to be divided into three clusters: low, medium, and high. The quality driver attributes were spread throughout all three clusters. High scoring wines have low frequency of citation for negative drivers and high for the positive, while the low scoring wines showed the opposite. Looking at the clusters formed by the wine samples in the same heatmap (Figure 3), some association with the quality scoring results could be made. One of the clusters corresponded to the seven highest scoring wines; the other two clusters were a mixture of the eight lowest scoring, but the clusters did not correspond to the statistical grouping from the ANOVA performed on the scores (Table S1).

**Figure 5:**
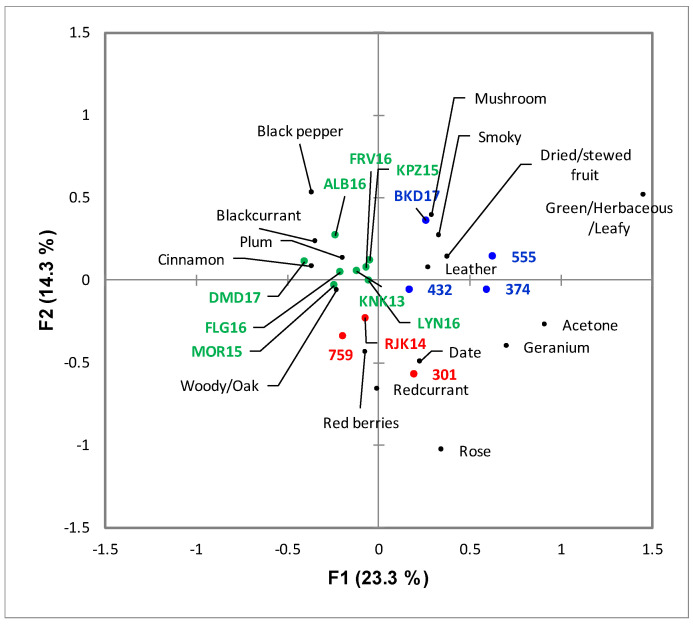
Correspondence Analysis biplot of the CATA results for the Pinotage wines illustrating the sensory space of the samples evaluated. The samples are color-coded according to the HCA clusters from Figure 3. Only significant attributes are represented (*p* < 0.05). The wine codes are as indicated in the text.

Only sixteen of the 47 attributes were found to be significant by the Cochran’s Q-test for the Pinotage set (Figure 5). The first two factors represented 23.3% and 14.3% of the inertia, totaling 37.6%. Considering the sensory space as represented in the CA biplot (Figure 5), the samples on the negative side of F1 corresponded to the higher scoring wines (Figure 1) and were associated with the positive quality drivers. On the other hand, the samples on the positive side of F1 correspond to the lower scoring wines (Figure 1) and were naturally associated with the negative drivers. However, the distribution of the wines in the sensory space was relatively tight and the samples tended to group close to the origin.

**Figure 6:**
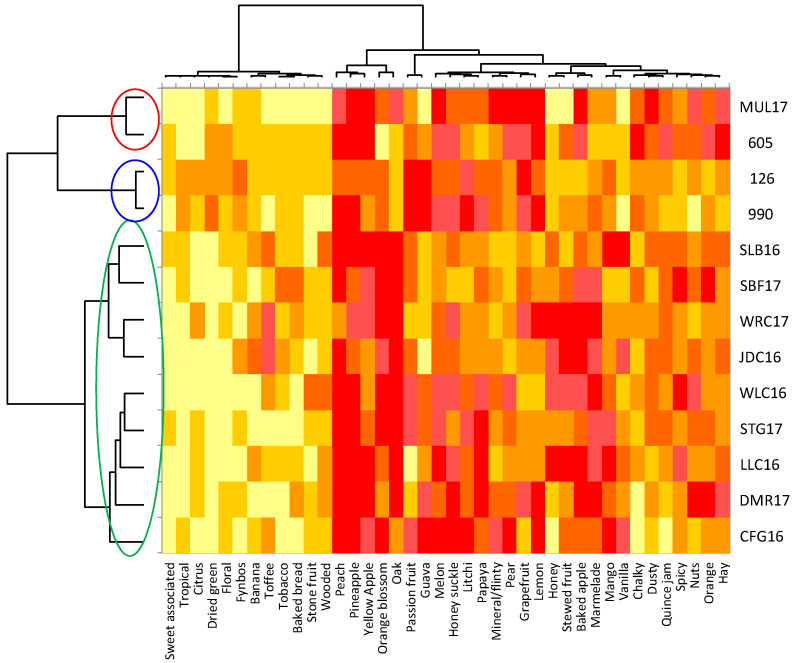
Heatmap associated with the results of the CATA task for the Chenin Blanc wines evaluated. The dendrogram on the left corresponds to the Hierarchical Cluster Analysis on the CATA results. Wine codes as indicated in the text.

**Figure 7:**
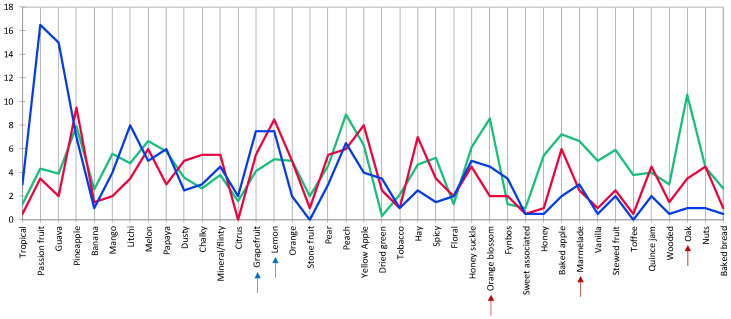
HCA cluster profile plot for Chenin Blanc. The blue and red arrows indicate negative and positive drivers of quality, respectively. The color of the graph lines corresponds to the clusters indicated in Figure 6.

The sensory space for the Chenin Blanc set as represented on the heatmap (Figure 6) presented a different configuration than for Pinotage. The dendrogram for the attributes and their frequency of citation was divided into two main clusters: low and high. The positive quality driver attributes were “marmalade”, “oak”, and “orange blossom”, and the negative ones were “lemon” and “grapefruit” (Figure 7). All driver attributes were in the high frequency cluster and visually they seemed to vary without an obvious trend. Considering the clusters formed by the wine samples (Figure 6), associations with the quality scoring results were evident: the three low scoring and one medium scoring wine (MUL17) formed one cluster, while the rest belonged to a different one. The sample with the score closest to MUL17 was WRC17 (Table S2), but in this analysis it was included in the same cluster as the high scoring wines.

**Figure 8:**
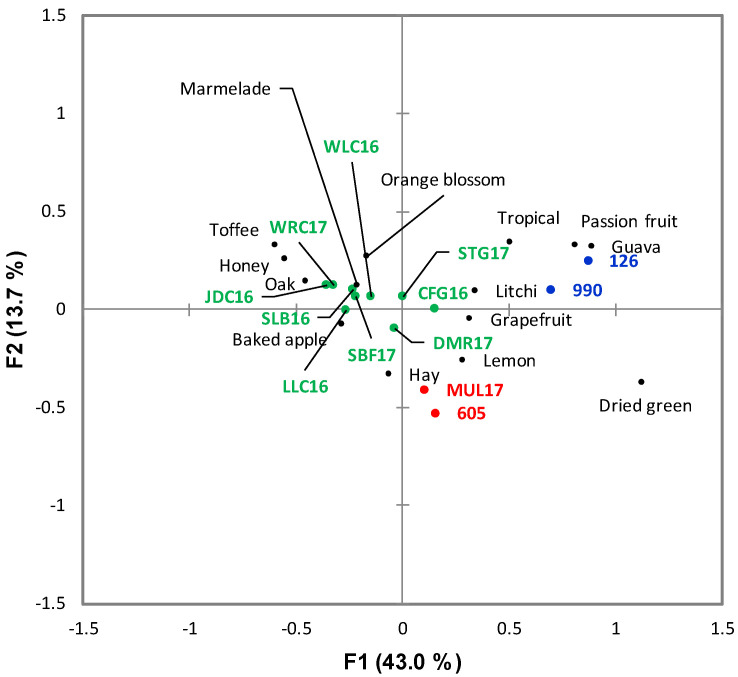
Correspondence Analysis biplot of the CATA results for the Chenin Blanc wines illustrating the sensory space of the samples evaluated. The samples are color-coded according to the HCA clusters from Figure 6. Only significant attributes are represented (*p* < 0.05). The wine codes are as indicated in the text.

Of the 39 attributes, fourteen were found to be significant for the Chenin Blanc according to the Cochran’s Q test (Figure 8). The first two factors represented 43.0% and 13.7% of the inertia, totaling 56.7%. The configuration of the sensory space was different from the one resulting from the quality score groups. The lowest scoring wines were found on the positive side of F1 together with a medium score wine (MUL17) and a high scoring one (CFG16), while another of the low scoring wines (WRC17) could be found on the negative side of F1 with the rest of the higher scoring wines. The two attributes determined as negative drivers of quality, “grapefruit” and “lemon”, were found on the positive side of F1, while the three positive drivers, “oak”, “marmalade”, and “orange blossom” were in the diagonally opposite quadrant. Contrary to Pinotage, none of the drivers were far from the origin. On both the CA, heatmap and HCA, winning wines clustered together with the exception of MUL17. Non-winning wines 990 and 126 clustered together and clearly showed nuances of “passion fruit” and “guava”. These attributes were not identified as negative drivers of quality, but without the presence of positive drivers of quality wines with these characteristics would not win a “Top 10 award”.

### 3.3. Chemical Space vs. Sensory Space

**Figure 9:**
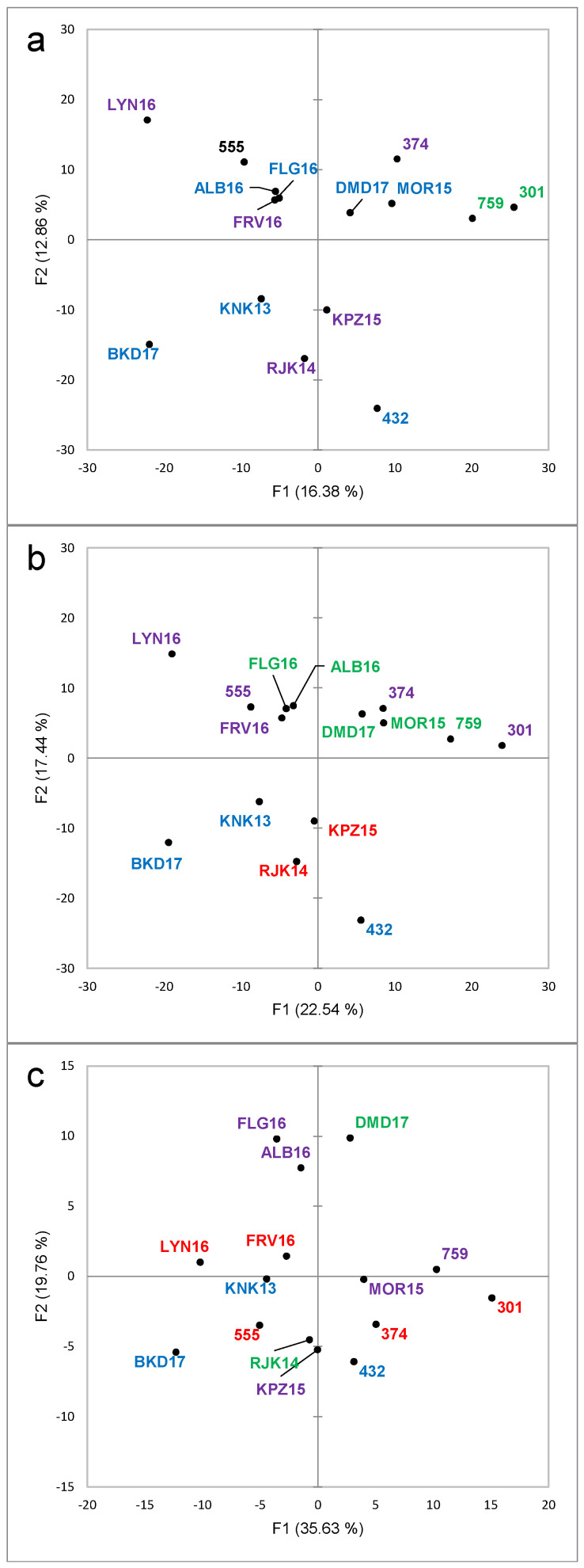
Principal Component Analysis score plot for Pinotage wines based on the results of the Liquid Chromatography-High Resolution Mass Spectrometry (LC-HRMS) analysis in positive and negative mode. Wine codes as indicated in the text. Groupings in the score plots are color-coded according to the Hierarchical Cluster Analysis on the same data. (**a**). Raw data; (**b**). First variable selection; (**c**). Second variable selection. Variable selection as described in the text.

**Figure 10:**
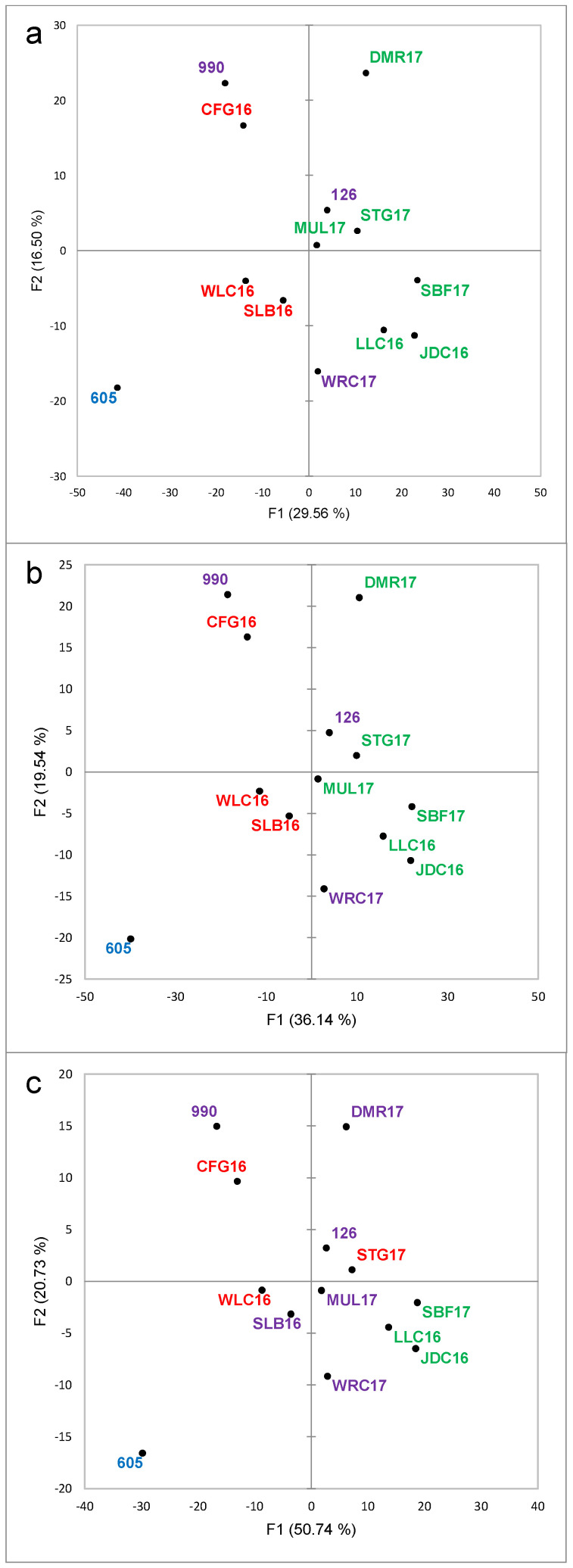
Principal Component Analysis score plot for Chenin Blanc wines based on the results of the LC-HRMS analysis in positive and negative mode. Wine codes as indicated in the text. Groupings in the score plots are color-coded according to the Hierarchical Cluster Analysis on the same data. (**a**). Raw data; (**b**). First variable selection; (**c**). Second variable selection. Variable selection as described in the text.

The results of the chemical analysis were subjected to the same type of unsupervised statistical analysis to determine possible natural groupings or clusters for the samples. In the case of Pinotage, the explained variance for the first two PCs was 16.4% and 12.9% (Figure 9a), while for Chenin Blanc, the first two PCs explained 29.6% and 16.5% of the variance (Figure 10a). These values would be considered generally low for chemistry data. However, in the case of fingerprinting methods, these low values are usual if no feature selection has been performed, as the noise included in the data is also higher than for targeted analyses [34]. The higher explained variance for Chenin Blanc could be due to the lower number of samples included in the sample set, thirteen compared to fifteen for Pinotage.

The 1466 MS features (RT_m/z signals) could be considered an advantage in terms of the amount of information obtained; however, the conundrum is this situation is that, with more information, more noise is also included in the data. Feature selection is supposed to considerably increase the ease of data interpretation. Two rounds of variable selection were performed, each of them consisting of PCA on the correlations matrix and selecting all the variables with the highest squared cosines (min 0.5) for the first three dimensions. As expected, the sample configuration was maintained while the explained variance increased as the noise decreased (Figures S1 and S2); *i.e.*, for the first two dimensions from 29.3% to 40% and 55.4% and from 46.1% to 55.7% and 71.5% for Pinotage and Chenin Blanc, respectively (Figure 9b–c and Figure 10b–c).

For both datasets, all clusters derived from the HCA (raw and selected variables) contained both Top 10 and low scoring wines (Figure 9 and Figure 10 and Figures S1 and S2). These clusters did not correspond to the groups from quality rating (Tables S1 and S2) or to the clusters from the sensory evaluation (Figure 3 and Figure 6).

No trends could be observed based on the loadings, even for the models generated after variable selection (Figures S1 and S2). The clear association of samples or clusters with specific ions could not be described as the chemical space the samples and clusters occupied was overlapping, according to the bootstrap ellipses (Figures S1 and S2).

The configurations resulting from the sensory and chemical data were compared pair-wise for each cultivar. The RV coefficients calculated from the first three dimensions of the CA and PCA were 0.444 and 0.511 for Pinotage and Chenin Blanc, respectively. Only in case the values are higher than 0.7 it can be considered that the configurations are similar.

## 4. Discussion

In view of the methodology aim of the work, it was demonstrated that combining CATA with the quality rating is a quick way of profiling the wines selected and determining their quality scores in a non-competition environment. Compared to the previous methodology proposed that combined sorting as a rapid method with quality rating [18], for the current methodology, the tasks were completed in one step and not in two. This approach also avoided the wines being presented to the judges once as a group and the second time monadically; the rating was carried out at the same time as the profiling for one wine at a time.

One of the challenges for CATA is the attributes list [18]. A relevant aspect to consider when compiling such a list that will eventually be used for correlation or comparison with quality rating is that the terms included must be linked to quality proxies [3]. The number and the nature of the attributes chosen also have to cover the possible sensory space of the wine set, which will be more complex if the samples are commercial and come from a variety of producers and styles. By choosing Chenin Blanc and Pinotage, the current study aimed to benefit off of the in-depth knowledge South African industry professionals have of these two iconic wines. Additionally, aroma wheels for both cultivars are available and were used in this study [32,33] and familiar to the judges, so in this case the choice of terms for the CATA list was straightforward.

It would be difficult to compare the performance of various methodologies unless the evaluations were completed on the same set and with the same aim. The choice of samples can affect the results of both quality rating and sensory evaluation through the attributes generated. When evaluating typicality (of a high quality wine in this case), various degrees of representativeness for the prototype are required [35,36] in order to have examples of both high quality and low (which in this case would constitute the borders of the concept of high quality). The choice of the researcher can be difficult; if the wines in the set are all considered representative of a category, region, or style, they might not be varied enough in terms of quality. The number of samples included in a set can also influence the outcome in terms of information generated, explained variance within the set, and the robustness of results. Studies approached these issues differently with various degrees of success. For the current study, the wines chosen were from local competitions; in addition to the Top 10 winners, low scoring wines were included as representatives of the boundaries of the high-quality concept. In the case of Californian Cabernet Sauvignon wines, the authors chose 27 wines from three quality categories according to competition results [4,11], while other authors opted for experimental wines considered free of faults as the simplest rule for quality [28,37] but with a higher number of wine samples (60 Cabernet Sauvignon and 50 Chardonnay wines) or even wines all with ratings higher than 90 points according to wine critics (83 Australian Chardonnay wines) [27]. In a previous study conducted in South Africa, only eight Sauvignon Blanc wines were included in the set, all chosen by industry professionals as “representing premium quality” [18]. However, in that case, the goal of the study was to propose a new methodology and the associated fast workflow appropriate to use in an industry context.

In the current study, other than the wine sensory characteristics, the difference in the numbers of wines in each sample set could have been one of the causes for the explained variance in the CA (higher for Chenin Blanc, a smaller sample set). The number of wines in a sample set also had to take into account the sensory tasks and the judges that performed them. Even though quality can be evaluated with consumers, the industry professionals were also chosen in the current study due to their familiarity with the method and the lexicon included in the CATA lists used. The judges are used to evaluate a large number of samples in one session, but the scope of this study was not known to them prior to the tasting session and what was asked of them was also different from competition conditions in terms of sample profiling.

The evaluation of wine quality (or of quality wines) is carried out with various goals in mind, using different types of judges and thus the sensory methods and the statistical data handling vary. The correlation of rating (for example of hedonic rating by consumers) with other properties of the product can be carried out by generating an external preference map [38,39]. It was proposed that if the rating is carried out by experts and it is aimed at quality, the same approach would be an external quality mapping [3]. These types of approaches are aimed ultimately at correlating quality rating with sensory attributes and/or generating drivers for quality and predicting the sensory attributes of a high quality wine using supervised statistical techniques [4,11,28,37]. In the case of Australian Sauvignon Blanc and Chardonnay, the wines were sorted into quality groups and described by DA; the statistical analyses consisted of CVA and MDS and the results were linked through GPA [37]. For Californian Cabernet Sauvignon wines, results generated through DA were subjected to PCA and the quality scores to DISTATIS; the correlation between these datasets was completed by PLS2 and cross-validated by leave-one-out procedure as the number of sample was limited [11]. When the aim of a study is exploring the sensory space of high-quality wines, the sensory methods and the statistical approach will differ. However, in the case of the current study, an unsupervised method such as CA was considered more appropriate for exploring the sensory space of the wine sets, then combined with the Pearson coefficient to determine the drivers for quality [18]. As in the cited work, the aim of the current study work was not to predict quality based on sensory data, but rather to elucidate the sensory drivers for quality for the specific set.

Seen as a much more objective way of characterizing a wine, chemical analysis is sometimes included in studies focused on wine quality. It is easy to see why certain classes of compounds would be related to quality through proxies—for example, volatile compounds contribute to aroma, polyphenols to color and taste. Normally, a limited number of compounds analyzed does not provide a comprehensive picture of the wine chemical space. An information-rich technique such as MS could be used to fingerprint the wines and appropriate statistical tools would reveal the compounds driving the quality. Even though in the current study, the same statistical approach was to be considered for the chemistry data as for the sensory results (calculating the Pearson’s correlation coefficients between the quality scores and the standardised deviates from the PCA), the chemical data proved to be too complex and the 1466 features included in the PCA contained a high level of noise. Even after feature selection, which led to noise reduction, this operation could not be performed. In the case of supervised statistical methods, feature selection is more straightforward as it leads to better separation, regression, etc.—aspects that have performance indicators that are easier to evaluate; conversely, the aim of the current work was to explore the space of the sample sets, and the statistical methods were unsupervised. Orthogonal PLS-Discriminant Analysis (OPLS-DA) and the S-plots associated could have been an option in the case of supervised modelling; even in that case, the very limited number of samples and the criteria for choice of classes (based on competition results, quality rating or sensory evaluation) would have made this analysis unadvisable. Another aspect of statistical relevance was that the matrix was not balanced, containing 13 and 15 samples (observations) for Chenin Blanc and Pinotage, respectively compared to the 1466 MS signals (variables). To obtain a more balanced matrix, the number of variables should be reduced and/or the number of samples should be increased considerably. One way is reducing the number of variables through statistical means, as completed in this study. Additionally, the number of variables can be limited a priori by targeting compounds (analytically), but this approach makes the assumption that the researcher would know which compounds are critical for the quality or the quality proxies. If the list of targeted chemical compounds contributing to the wine quality is comprehensive, the methodology would correspond to a targeted metabolomics approach, but even in that case, the same assumption is made even if not to the same extent. The alternative, increasing the number of samples, was impossible in the circumstances of the current study. If the same type of study were to be carried out over a number of years, the chances of success for the statistical analysis would increase. However, one should consider that the style of winning wines might change in time, that the panel used for quality evaluation (during and outside the competition) would change, and even the palate of the judges would change.

Therefore, the sample configurations (score plots) derived from the PCA on the MS data were considered sufficient in the current study, as they allowed for the comparison of sensory and chemistry spaces for each dataset through RV coefficients. This type of approach is not usual in literature related to wine quality. Previous works reported on the correlation between quality proxies such as judgement points and/or expert scores and a limited number of individual aroma compounds or even chemical elements; in the latter case, the more likely explanation was that the elements were rather markers for the origin of the wine and thus, indirectly, the authors linked the quality indicators to the wine origin, which they already regarded as a quality proxy [4]. In the same study, the chemical profile obtained by HS-SPME-GC-MS for 64 volatiles and the sensory profile by DA were submitted separately to PCA and then compared pair-wise through Pearson’s product correlation coefficient, showing both positive and negative correlations between compounds and aromas. For Australian Chardonnay, 83 wines were scored on a 20-point scale and chemically analyzed for 39 volatiles by HS-SPME-GC-MS; the two datasets were correlated through PLS [27].

## 5. Conclusions

Even though quality is not a well-defined concept and a complicated topic to tackle, it is interesting and can be an opportunity to develop new methodologies. The study demonstrated that, using the appropriate experimental conditions and statistical tools, it was possible to explore the sensory space of quality wines and to determine the sensory drivers for quality (positive and negative) as evaluated by industry experts. The idea of obtaining a detailed chemical fingerprint of the wines in order to determine the chemical drivers for quality in a similar manner as for sensory was interesting and worth exploring. However, in practice, the identification of chemical drivers (ions/compounds) was challenging due to the number of signals generated by the information-rich technique chosen compared to the number of samples included in the set.

The results of this type of investigation could be used to explore the sensory space of a wine sample set with the added dimension of the quality drivers which, in turn, highlight the experts’ opinions on what makes a winning wine. A consistent investigation of this kind can lead, over the years, to the elucidation of the trends in winemaking styles and in the professionals’ opinions, which in turn influence the following years’ styles.

## Figures and Tables

**Table 1:** Samples and sample codes of the wines included in the current study.

Wine	Wine Code
Pinotage Top 10 winners	
Allée Bleue Black Series Old Vine 2016	ALB16
Beyerskloof Diesel 2015	BKD17
Diemersdal Reserve 2017	DMD17
Fairview Primo 2016	FRV16
Flagstone Writer’s Block 2016	FLG16
Kaapzicht Steytler 2015	KPZ15
Kanonkop 2013	KNK13
Lyngrove Platinum 2016	LYN16
Môreson The Widow Maker 2015	MOR15
Rijk’s Reserve 2014	RJK14
Pinotage low scoring wines	
Pinotage 1	432
Pinotage 2	374
Pinotage 3	555
Pinotage 4	759
Pinotage 5	301
Chenin Blanc Top 10 winners	
Cederberg Private Cellar Five Generations 2016	CFG16
DeMorgenzon Reserve 2017	DMR17
Jean Daneel Wines Signature 2016	JDC16
Leopard’s Leap Culinaria 2016	LLC16
Mulderbosch Vineyards Steen op Hout 2017	MUL17
Slanghoek Wynkelder Legends Barrel Fermented 2016	SLB16
Spier Wines 21 Gables 2017	STG17
Stellenrust ‘53′ Barrel Fermented 2017	SBF17
Wellington Wines La Cave 2016	WLC16
Wildekrans Barrel Select Reserve 2017	WRC17
Chenin Blanc low scoring wines	
Chenin Blanc 1	990
Chenin Blanc 2	126
Chenin Blanc 3	605

## Supplementary Materials

The following are available online at https://www.mdpi.com/2304-8158/9/6/805/s1, Table S1: Descriptive statistics and ANOVA results for the Pinotage wines evaluated. The letters denote significance groups (*p* < 0.05). The codes are as specified in the text, Table S2: Descriptive statistics and ANOVA results for the Pinotage wines evaluated. The letters denote significance groups (*p* < 0.05). The codes are as specified in the text; Supplementary Figure S1. PCA loadings (1), biplots (2) and dendrograms (3) for the Pinotage LC-HRMS data based on a. raw data, b. first variable selection, c. second variable selection. Variable selection as described in the text; Supplementary Figure S2. PCA loadings (1), biplots (2) and dendrograms (3) for the Chenin Blanc LC-HRMS data based on a. raw data, b. first variable selection, c. second variable selection. Variable selection as described in the text.
