# Supplementary material for: Wine Quality Drivers: A Case Study on South African Chenin Blanc and Pinotage Wines
Source: Foods. 2020 Jun 18;9(6):805. doi: 10.3390/foods9060805 (PMC7353515; doi:10.3390/foods9060805)
Supplement: Supplementary file 1 [file foods-09-00805-s001.pdf]

Table S1. Descriptive statistics and ANOVA results for the Pinotage wines evaluated. The letters denote significance groups (P < 0.05). The codes are as specified in the text.

Wine Code	LS means	Standard error	Lower bound (95%)	Upper bound (95%)	Groups				
KNK13	15.789	0.457	14.889	16.690	A				
DMD17	15.667	0.470	14.742	16.592	A				
FLG16	15.632	0.457	14.731	16.532	A				
MOR15	15.474	0.457	14.574	16.374	A				
ALB16	15.412	0.483	14.460	16.363	A				
FRV16	15.211	0.457	14.310	16.111	A	B			
KPZ15	15.105	0.457	14.205	16.005	A	B	C		
RJK14	15.053	0.457	14.152	15.953	A	B	C		
759	14.895	0.457	13.995	15.795	A	B	C		
LYN16	14.737	0.457	13.837	15.637	A	B	C	D	
BKD17	14.053	0.457	13.152	14.953		B	C	D	
301	13.889	0.470	12.964	14.814			C	D	
432	13.526	0.457	12.626	14.426				D	E
374	13.500	0.470	12.575	14.425				D	E
555	12.421	0.457	11.521	13.321					E

Table S2. Descriptive statistics and ANOVA results for the Chenin Blanc wines evaluated. The letters denote significance groups (P < 0.05). The codes are as specified in the text.

Wine Code	LS means	Standard error	Lower bound (95%)	Upper bound (95%)	Groups			
SBF17	15.889	0.417	15.068	16.710	A			
STG17	15.556	0.417	14.734	16.377	A			
WLC16	15.385	0.425	14.548	16.221	A	B		
CFG16	15.296	0.417	14.475	16.117	A	B		
DMR17	15.185	0.417	14.364	16.006	A	B		
LLC16	14.926	0.417	14.105	15.747	A	B	C	
SLB16	14.926	0.417	14.105	15.747	A	B	C	
JDC16	14.741	0.417	13.920	15.562	A	B	C	
MUL17	14.296	0.417	13.475	15.117		B	C	
WRC17	14.259	0.417	13.438	15.080		B	C	
126	13.815	0.417	12.994	14.636			C	D
990	13.778	0.417	12.957	14.599			C	D
605	12.926	0.417	12.105	13.747				D

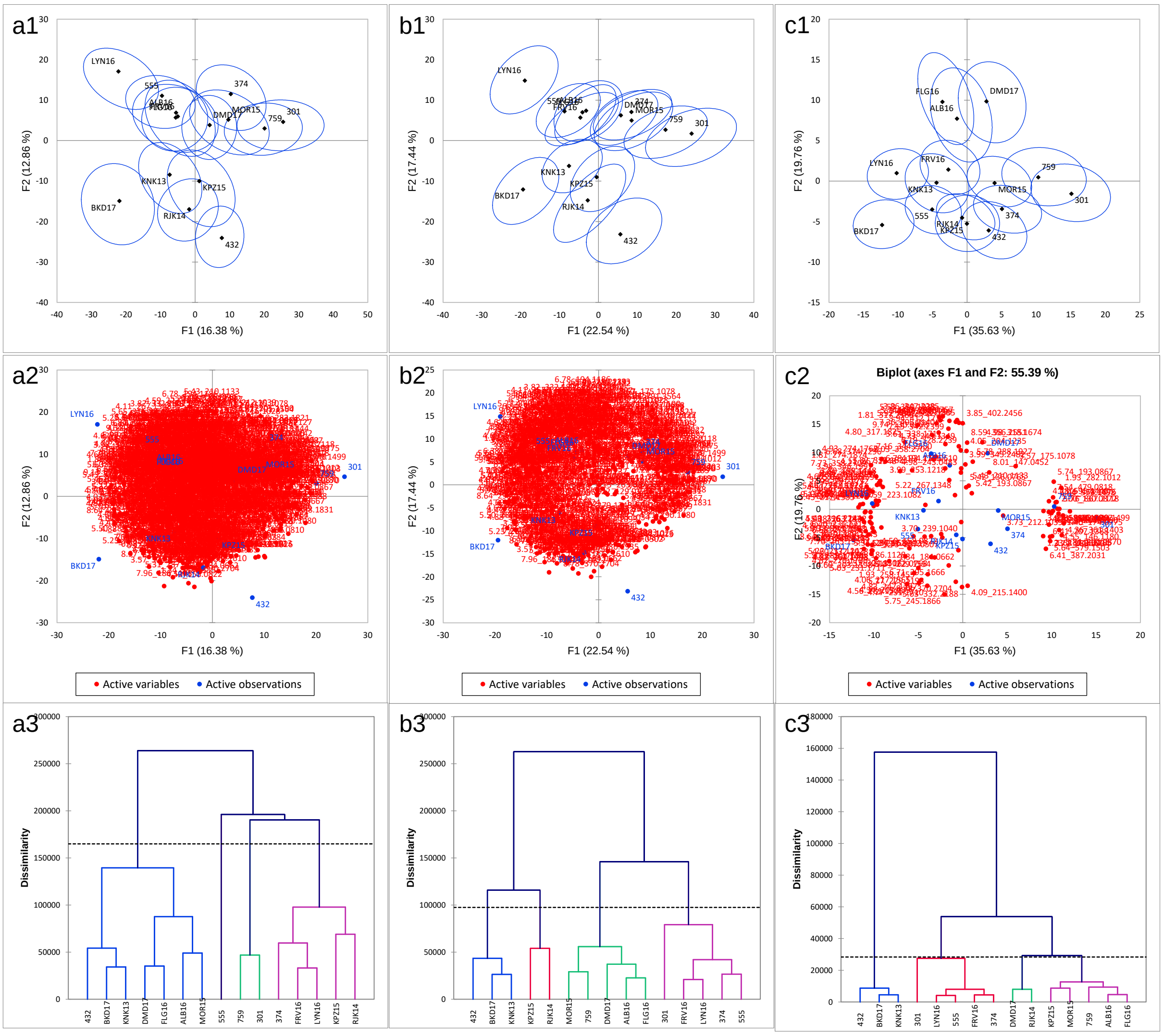

Figure S1. PCA loadings (1), biplots (2) and dendrograms (3) for the Pinotage LC-HRMS data based on a. raw data, b. first variable selection, c. second variable selection. Variable selection as described in the text.

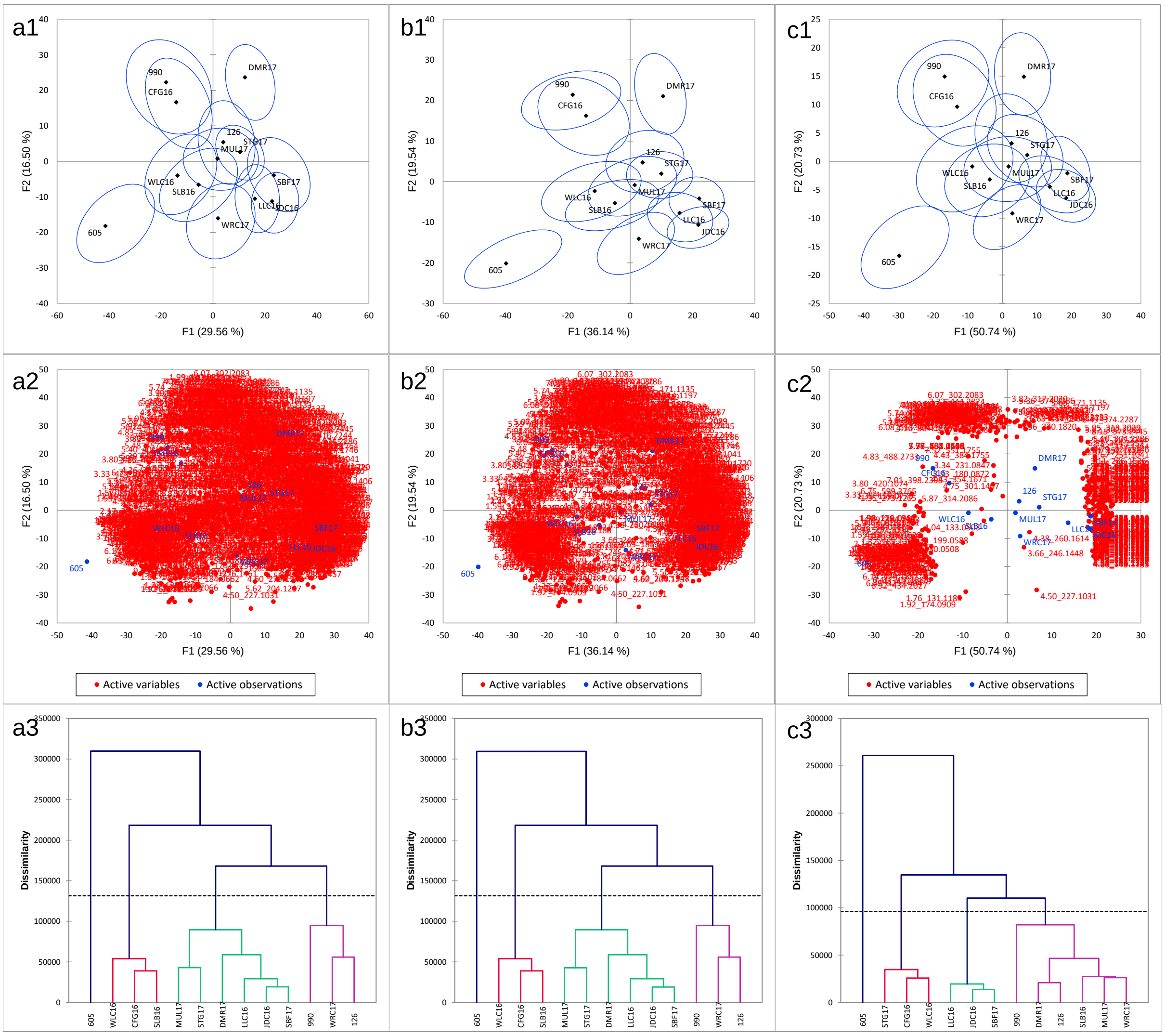

Figure S2. PCA loadings (1), biplots (2) and dendrograms (3) for the Chenin Blanc LC-HRMS data based on a. raw data, b. first variable selection, c. second variable selection. Variable selection as described in the text.
